# Can exercise benefits be harnessed with drugs? A new way to combat neurodegenerative diseases by boosting neurogenesis

**DOI:** 10.1186/s40035-024-00428-7

**Published:** 2024-07-25

**Authors:** Renqing Zhao

**Affiliations:** College of Physical Education, Yangzhou University, 88 South Daxue Road, Yangzhou, 225009 China

**Keywords:** Exercise mimetics, Neurological diseases, Neurogenesis, Cognition

## Abstract

Adult hippocampal neurogenesis (AHN) is affected by multiple factors, such as enriched environment, exercise, ageing, and neurodegenerative disorders. Neurodegenerative disorders can impair AHN, leading to progressive neuronal loss and cognitive decline. Compelling evidence suggests that individuals engaged in regular exercise exhibit higher production of proteins that are essential for AHN and memory. Interestingly, specific molecules that mediate the effects of exercise have shown effectiveness in promoting AHN and cognition in different transgenic animal models. Despite these advancements, the precise mechanisms by which exercise mimetics induce AHN remain partially understood. Recently, some novel exercise molecules have been tested and the underlying mechanisms have been proposed, involving intercommunications between multiple organs such as muscle-brain crosstalk, liver-brain crosstalk, and gut-brain crosstalk. In this review, we will discuss the current evidence regarding the effects and potential mechanisms of exercise mimetics on AHN and cognition in various neurological disorders. Opportunities, challenges, and future directions in this research field are also discussed.

## Introduction

With the ageing of society, the prevalence of neurodegenerative disorders, such as Alzheimer's disease (AD), Parkinson's disease (PD), and Huntington's disease (HD), is rapidly increasing [1–5], but effective treatments for these diseases are currently lacking. Physical exercise has long been known to have multiple benefits for brain health, including enhancing cognitive function, memory, learning, and attention [6, 7]. These benefits are believed to be linked in part to increased adult hippocampal neurogenesis (AHN) [8–10]. The subgranular zone of the hippocampal dentate gyrus (DG) contains neural stem cells (NSCs) that continuously produce dentate granule cells (DGCs) across the mammalian lifespan [11]. The process of AHN is primarily driven by experience, allowing the brain to adapt to environmental demands and affecting pre-existing circuitry and hippocampus-dependent memory function. Recent evidence from postmortem brain specimens revealed that AHN is persistent throughout life in humans, and its magnitude is affected by the presence of a variety of neurodegenerative diseases, such as PD, AD, and HD [12–15]. Interestingly, a recent study applied the single nucleus RNA sequencing (snRNA-seq) technique to profile neurogenic trajectory in the adult human hippocampus and revealed persistence of neurogenesis in humans across divergent ages [16, 17]. In addition, the percentage of immature granule cells is lower in AD patients than that in matched controls [16]. Increasing evidence has indicated that AHN is impaired in various neurodegenerative conditions such as AD, PD, and HD [18, 19]. While those diseases are not exclusively hippocampal disorders, they frequently affect the hippocampus and lead to cognitive symptoms [20, 21]. For example, the hippocampus is one of the earliest regions affected by dispersed aggregates of amyloid beta (Aβ) and tau proteins, leading to impaired hippocampal neurogenesis and memory deficits in AD [20, 21]. Enhancing neurogenesis in the hippocampus, such as through reduction of tau [22] and APP accumulation [23] and increment of neurotrophic factors [24], could potentially mitigate the adverse effects of AD pathology on the hippocampus. This may offer a compensatory mechanism to counteract cognitive dysfunction, the loss of neuronal connectivity, and cell death associated with AD [20, 25]. Similarly, the hippocampus is vulnerable to the accumulation of fibril α-synuclein, resulting in non-motor symptoms such as cognitive decline and mood disorders in PD [26, 27]. HD is characterised by cognitive deficits and affective disorders [28], and hippocampal disorders are involved in the cognitive and mood symptoms associated with HD [29]. Targeting AHN through interventions such as environmental enrichment and exercise might counteract the negative impacts of neurodegenerative conditions on the hippocampus, resulting in improved memory processing, reduced depression, and enhanced brain plasticity in PD [27, 30] and HD [31, 32]. Taken together, as the hippocampus is a common area impaired in neurodegenerative conditions, a growing body of evidence has suggested the potential of AHN-targeting strategies for neurodegenerative conditions [20, 25, 27, 30–32].

Physical exercise promotes adult neurogenesis, modifies synaptic connections, and enhances cognitive functions by activating a variety of molecular and cellular processes [33–35]. Interestingly, the exercise-induced pro-neurogenic effects have been replicated through administration of certain compounds such as brain-derived neurotrophic factor (BDNF), irisin, and clusterin derived from ‘runner plasma’, which boost neurogenesis and enhance cognitive function in rodents [34, 36, 37]. The concept of ‘exercise mimetics’ indeed has attracted much interest; it represents a new approach to ameliorating neurodegeneration and improving cognitive function in ageing and neurodegenerative disorders by replicating, to some extent, the effects of exercise [38, 39]. It is particularly important for the elderly or the diseased people who have difficulty in conducting regular exercises. Additionally, evidence indicates that the newborn neurons do not always work efficiently with local neural networks; instead, they induce disturbance in the existing circuits, resulting in forgetting. This disturbing impact could be ameliorated by exercise intervention [40], indicating that exercise mimetics may also facilitate the integration of newly generated neurons into the existing circuitry.

Significant progresses have been made in the regulation of adult neurogenesis, such as using pro-neurogenic factors to promote neurogenesis, re-activation of endogenous neurogenesis or directed reprogramming in situ. Among various strategies, targeting AHN by administering specific compounds that replicate the effects of exercise is a promising new approach [34, 36, 37]. This review aims to address critical questions and emerging evidence related to the impact of exercise mimetics on adult neurogenesis and its relevance in neurodegenerative disorders. Particularly, the following aspects are focused on in this review: (i) the generation and modulation of adult neurogenesis and its relevance in neurodegenerative diseases; (ii) the effects of exercise mimetics on AHN and their potential benefits in animal models and patients with brain disorders; (iii) the mechanisms underlying the action of exercise mimetics; and (iv) the challenges and future directions of putative molecules of exercise mimetics.

## Neurogenic deficit is an essential feature of neurodegeneration

Impaired hippocampal neurogenesis is an essential feature of neurodegeneration in both ageing and diseases. The ageing-associated neurodegeneration is characterised by a decline in AHN and impairment of cognition and memory [2]. Aged mice show a significant reduction of neurogenesis in both the subventricular zone and DG niches, leading to a marked decrease of newborn neurons [41]. Similar to rodents, decreased hippocampal neurogenesis has also been observed in aged humans. One study examined doublecortin (DCX)-positive immature neurons in 13 healthy individuals aged between 43 and 87 years, and showed decreased number of DCX-positive cells in the DG of older adults [12]. Older individuals also have a smaller quiescent NSC pool in the DG and reduced neuroplasticity and angiogenesis [14]. Neuroinflammation in the aged central nervous system (CNS) may contribute to reduced neurogenesis [42]. Accordingly, exposure of older animals to the young blood can restore neurogenesis and cognition [43]. Additionally, the maintenance of neurogenesis depends on a closely integrated network of signals, and changes to these signalling pathways during ageing have been associated with the reduction in neurogenesis [44].

The neurogenic niche is susceptible to neurodegenerative conditions [45, 46]. Neuroinflammation is one of the key features of neurodegenerative disorders, such as AD, PD, and HD. It can be stimulated by Aβ plaques, fibrillar tau, or α-synuclein aggregates [45, 46]. Postmortem brain tissue analysis of PD patients revealed hippocampal atrophy, reduced NSCs in the DG region, as well as an increased number of reactive microglia and elevated levels of pro-inflammatory factors, such as tumor necrosis factor-alpha (TNF-α), interleukin (IL)-1β, and IL-6 in the midbrain and cerebrospinal fluid [47]. Moreover, a recent study examining postmortem brain samples of 45 AD patients aged 52 to 97 years suggested a decreased number of immature neurons at all stages of the disease compared to healthy individuals of similar age [12]. Similarly, transgenic AD models, such as the 3 × Tg, 5 × FAD, and APP overexpression models, show a clear neurogenic impairment [21]. HD also shows obvious AHN deficits. In HD patients, newly generated neurons display early maturation impairments, morphological abnormalities, and reduced expression of NeuN [13], consistent with the observed impairments in AHN in transgenic HD mouse models [48]. DG astrogliosis and elevated microglial activations are recognized as detrimental to the homeostasis of the DG neurogenic niche [13]. Together, neurodegenerative conditions have a profound impact on AHN and memory function, which also offer a potential therapeutic strategy for neurodegenerative disorders, i.e., boosting neurogenesis by exercise and agents mimicking the effects of exercise. The correlation between AHN and neurodegenerative diseases and the potential therapeutic approach will be discussed in the sections below.

## AHN shapes the plasticity of mammal brains

**Fig. 1:**
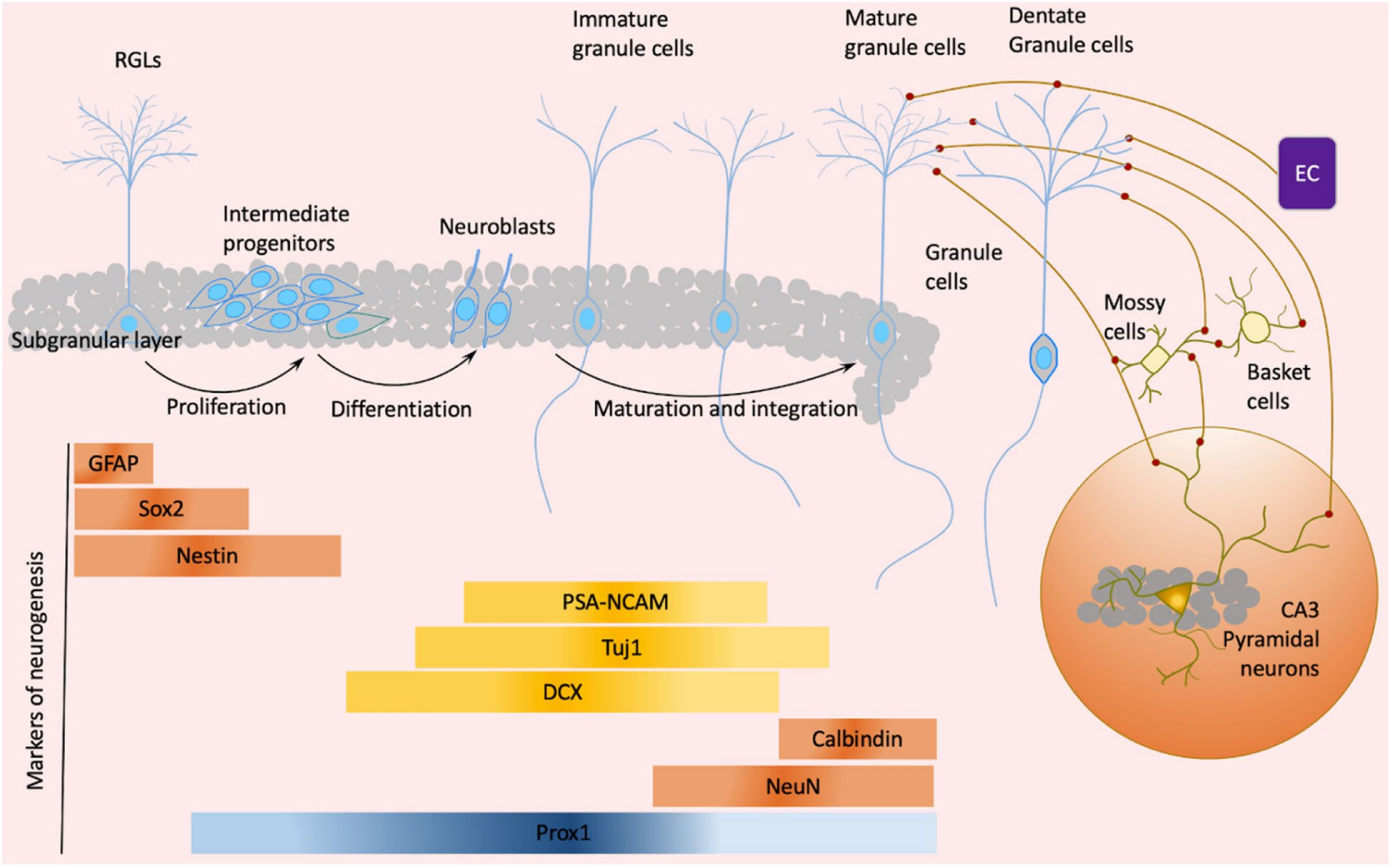
Proliferation, division, maturation, and integration of adult hippocampal neurogenesis in mammalians. Radial glia-like neural stem cells (RGLs) reside in the subgranular zone (SGZ) of the hippocampal dentate gyrus (DG) and give rise to new granule cells. RGLs can be activated and differentiate into dentate granule cells (DGCs) in response to neurogenic niche signals, making subsequent fate choices: (i) self-replicate to expand RGL pools; (ii) generate neurons; (iii) generate one RGL and one non-RGL; (iv) divide into two non-RGLs; (v) give rise to astrocytes [382]. Activated RGLs experience remarkable genetic and metabolic changes, such as upregulated expression of achaete-scute homologue (ASCL1), and cell metabolism-associated cell activation [383, 384]. Adult-born neurons in the rodent hippocampal DG ultimately develop into excitatory, glutamatergic granule neurons within the following several weeks after birth. However, not all newly generated DGCs in rodents can survive; finally, a substantial fraction of newborn DGCs die within three weeks after division, leaving less than one-quarter of newborn neurons to survive and integrate into the existing circuits [55, 56]. EC, Entorhinal cortex; GFAP, Glial fibrillary acidic protein; DCX, Doublecortin; NeuN, Neuronal nuclei; Prox1, Prospero homeobox protein 1; PSA-NCAM, Polysialylated-neural cell adhesion molecule

Over the past two decades, significant progress has been made in understanding the origin, formation, regulation, and integration of adult-born DGCs in the mammalian brain [49, 50]. NSCs serve as the main origin of new hippocampal neurons in the adult brain. They are located in the neurogenic niche, an area enriched with various types of cells, extracellular matrix, and cortical and subcortical neuronal projections, which collectively provide diverse signals essential for maintaining the function of NSCs [51] (Fig. 1). NSCs reside mainly in a dormant, quiescent state, and the quiescent NSCs proliferate as an adaption to environmental cues such as feeding and exercise [52–54]. Dormant NSCs can be activated and differentiate into DGCs in response to changes of neurogenic niche signals. During integration and maturation, approximately 75% of newborn granule cells will die within three weeks after division [55, 56]. The integration of adult-born neurons into the hippocampal circuit is accomplished by their competition with mature granule cells for the perforant pathway input. Newborn neurons with more spines may undergo an accelerated integration process [57–59]. Those circuits connecting anatomically and physiologically between the entorhinal cortex, DG, and CA3 facilitate the resolution of memory interference.

The newly generated neurons are critical for the resolution of memory interference by improving hippocampal memory discrimination, consolidation, and clearance. Depleting hippocampal neurogenesis increases memory interference during reversal learning tests [60–62], whereas genetically expanding the number of newborn DGCs had the opposite effect [59]. Newly generated DGCs are also crucial for expediting the consolidation of prior experiences and improving the precision of long-term memory [61, 63, 64]. As a result, they facilitate discrimination between subsequent experiences. Furthermore, the ongoing formation and integration of newly generated neurons into the preexisting circuitry facilitate a unique memory phenomenon called ‘active forgetting’. This process is essential for memory encoding and consolidation by degrading or eliminating existing information stored in hippocampal circuits, thereby creating space for new learning [40, 65]. However, AHN is often impaired in various neurological disorders. It can also be enhanced by favourable conditions such as exercise. Therefore, AHN may hold the potential as a therapeutic target for many neurodegenerative diseases.

## New evidence for the presence of AHN in humans

The existence of AHN has been validated in non-human mammalians, but remains debated in humans since the first report of hippocampal neurogenesis in rodents [11]. Eriksson et al*.* [66] led pioneering work to reveal the existence of adult neurogenesis in the human hippocampus by incorporating a thymidine analogue, bromodeoxyuridine (BrdU), into the DNA of cells undergoing division. Later, another prominent work conducted by Spalding et al*.* [67], measured the immediate increment of incorporated ^14^C in dividing cells, and validated the presence of adult-born granule cells in the human hippocampus. Following those studies, a growing range of studies extended the evidence to support the existence of adult neurogenesis in humans [68–75].

However, there are still discrepancies in the findings related to this phenomenon [12–14, 76–78]. Recent emerging techniques, such as snRNA-seq analysis, have brought a novel perspective on this issue. Habib et al*.* [79] first generated high-quality single-nuclei transcriptomic profiles from preserved adult human postmortem tissues and detected a cluster of 201 hippocampal cells identified as NSCs based upon known marker gene expression [79]. Subsequently, two research groups applying snRNA-seq analysis [16, 17], revealed the cell populations, transcriptomic markers, and neurogenic trajectory within the adult human hippocampus. Interestingly, one study [16] utilized an enhanced snRNA-seq protocol, incorporating a machine learning-based analysis program, to reanalyse several previously published snRNA-seq datasets [79–81]. This study confirmed the presence of immature granule cells in all three snRNA-seq datasets, including the dataset [81] that had originally reported an extremely low rate of neurogenesis. Moreover, Zhou et al*.* [16] assessed the impact of neurodegenerative diseases on hippocampal neurogenesis and found that the number of newborn neurons was reduced by 50% in patients with AD compared to control subjects. Together, by applying advanced analysis approaches, researchers have now been able to better delineate NSCs, progenitors and immature neurons in postmortem human brain samples and reason out their neurogenic lineage and maturation. However, the snRNA-seq data are extremely scarce in human studies. To fully understand the neurogenic profiles associated with various neurodegenerative disorders, more studies are urgently needed.

## Neurodegenerative diseases and AHN: evidence and therapeutic implications

### AD and hippocampal neurogenesis

Individuals with AD exhibit a marked reduction in the number of viable precursor cells within the hippocampus [82]. Musashi-1- and Ki-67-positive precursor cells from AD patients are capable of self-renewal but reach senescence early [82]. Moreover, a recent study analyzed the number of adult-born neurons among 45 AD autopsy brain samples from subjects aged 52–97 years. The results showed that in contrast to the detection of thousands of immature neurons in the hippocampal DG of healthy subjects across the age, the number and the maturation of those cells markedly decreased in AD patients [12]. Additionally, in the study by Zhou et al*.* [16], the impact of neurodegenerative diseases on AHN was examined by snRNA-seq analysis on postmortem brain samples from AD patients. The findings indicated a 50% reduction in the proportion of immature granule cells in AD patients compared to the control group. Our current understanding of the mechanism of how AD pathology affects AHN is mainly based on the evidence from various animal models. 3 × Tg-AD mice, a most popular AD model carrying *APP*, *PSEN1* M146V, and *MAPT* P301L transgenes [83], display obvious protein clumps, tangles, neuroinflammation, cognitive dysfunction, and impaired hippocampal neurogenesis at an early stage (4 months) [84–86]. In an alternative transgenic rodent model of tauopathy, which specifically overexpresses human tau (htau) in gamma-aminobutyric acid (GABAergic) interneurons in DG, the hippocampal neurogenesis and morphology of immature neurons are impaired via suppression of GABAergic transmission and disruption of local neural circuitry [87]. Importantly, increasing GABAergic transmission by THIP restores hippocampal neurogenesis and improves cognitive function, providing a potential treatment strategy for AD [87]. Taken together, these studies indicate the vulnerability of the neurogenic niche and the maturation processes of newborn neurons in response to AD pathology.

### PD and hippocampal neurogenesis

Generally, postmortem analysis data on adult neurogenesis with respect to PD pathology are limited. Terreros-Roncal and colleagues [13] demonstrated that PD patients display an increased density of radial glia-like cells with NSC properties but abnormal morphology of DCX-positive granule cells with impaired maturation. Hoglinger et al*.* [88] determined the cells expressing nestin and β-III-tubulin in postmortem hippocampal DG from PD patients, and they showed that those immature neuronal markers were significantly down-regulated in the hippocampal DG compared to controls. A pathological hallmark of PD is the deposits of misfolded α-synuclein into Lewy bodies, which are cytotoxic to neural cells [89]. Winner et al*.* [90] generated α/β-synuclein double knockout (KO) murine models and detected a significant increment in newborn neurons in the hippocampus. Subsequently, in transgenic mice overexpressing human wild-type α-synuclein (Hwt-α-syn) under the control of platelet-derived growth factor-β promoter, they found reduced AHN and elevated cell death as well as impaired dendrite outgrowth, decreased dendritic length and lesioned dendritic branching in immature newborn neurons [90, 91]. Meanwhile, *Lrrk2* transgenic rodents expressing mutant G2019S display enhanced anxiety-associated behaviours and decreased hippocampal neurogenesis, leading to a 77% reduction in the number of immature adult-born neurons [92]. Generally, regional and cell-specific overexpression of α-synuclein or mutant *Lrrk2* results in the degeneration of selective circuitries, as well as motor deficits and inclusion formation featured in PD, ultimately leading to impairment of proliferation, differentiation and survival of newborn neurons [93–95]. Collectively, evidence from both clinical data and animal models of PD indicates that impaired AHN is a hallmark of PD pathology.

### HD and hippocampal neurogenesis

Early maturation defects, morphological changes, and reduced expression of NeuN have been detected in newborn neurons in the postmortem brain samples of HD patients [13]. DG astrogliosis and increased microglial activation may disrupt the homeostasis of the DG neurogenic niche. R6 lines are the most widely used HD transgenic animal models carrying 115 (R6/1) or 150 (R6/2) CAG repeats via introducing exon 1 of the human HD gene into the rodent germline [32, 96]. The R6/1 mice show a remarkable decrease (64%) in the number of newborn granule cells at age 20 weeks [96]. Moreover, both differentiation and survival of newborn neurons in the hippocampus are severely impaired in these mice, leading to an overall decrease in the population of mature newborn neurons [96]. In contrast, the R6/2 mice exhibit impaired hippocampal neurogenesis at an early stage (about 2 weeks) and a 70% decrease in the number of newly generated neuroblasts at 12 weeks of age [32]. Additionally, both R6 lines showed decreased expression of polysialylated-neural cell adhesion molecule in the hippocampus, indicating impairment in synaptic plasticity, axonal growth, dendritic branching and cell migration [97, 98]. Consistently, in another HD transgenic model, the YAC128 mice, newly generated granule cells in the DG are particularly impaired [99]. The decrease of neurogenesis is persistent from three months (early symptom) to 18 months of age (end-stage) compared to WT controls [99].

Together, the persistent decrease in hippocampal neurogenesis represents a featured neuropathological process in AD, PD, and HD. Given that hippocampal neurogenesis plays a critical role in hippocampal-dependent memory function, the impairment in neurogenesis might account for the cognitive dysfunction in those transgenic rodents.

## Exercise: an approach to combating neurodegeneration and boosting neurogenesis

### Exercise prevents neurodegenerative disorders in human studies

Neurodegenerative disorders are a group of diseases characterized by progressive loss of neurons and cognitive impairment, leading to disability and death [5, 100, 101]. These diseases pose a significant challenge to public health and social care for there is currently no effective treatment for those diseases. Exercise is a well-established therapeutic strategy to promote brain health and prevent the onset and progression of neurodegenerative disorders [6, 102, 103]. The latest report on AD in 2022 has proposed a range of risk factors for AD, particularly the lack of physical activity [104]. Accordingly, a large-scale study involving 160,000 participants revealed that regular exercisers have a 45% lower chance of developing AD [105]; a similar finding (with a 53% reduction in AD risk) was reported in a longitudinal study of 716 older adults on the association of physical activity with dementia status [106]. Moreover, a recent meta-analysis incorporating eight prospective studies with 544,336 participants and 2192 patients with PD evaluated the effect of physical activity on the risk of PD [107]. The results showed that physical activity, particularly moderate to vigorous physical activity, is associated with a significant reduction in PD risk (relative risk, 0.71; 95% CI, 0.58–0.87) [107], with a stronger association among men than women. Additionally, a Cochrane review provided robust evidence of beneficial effects of most types of physical exercise on the severity of motor signs and quality of life in people with PD, compared to the control group [108]. However, the results also revealed little evidence of differences between intervention protocols, leading to the difficulty of recommending the best type of exercise for individuals [108]. Collectively, the current findings have provided robust evidence for the preventive role of physical exercise in neurodegenerative disorders [102, 109, 110].

**Fig. 2:**
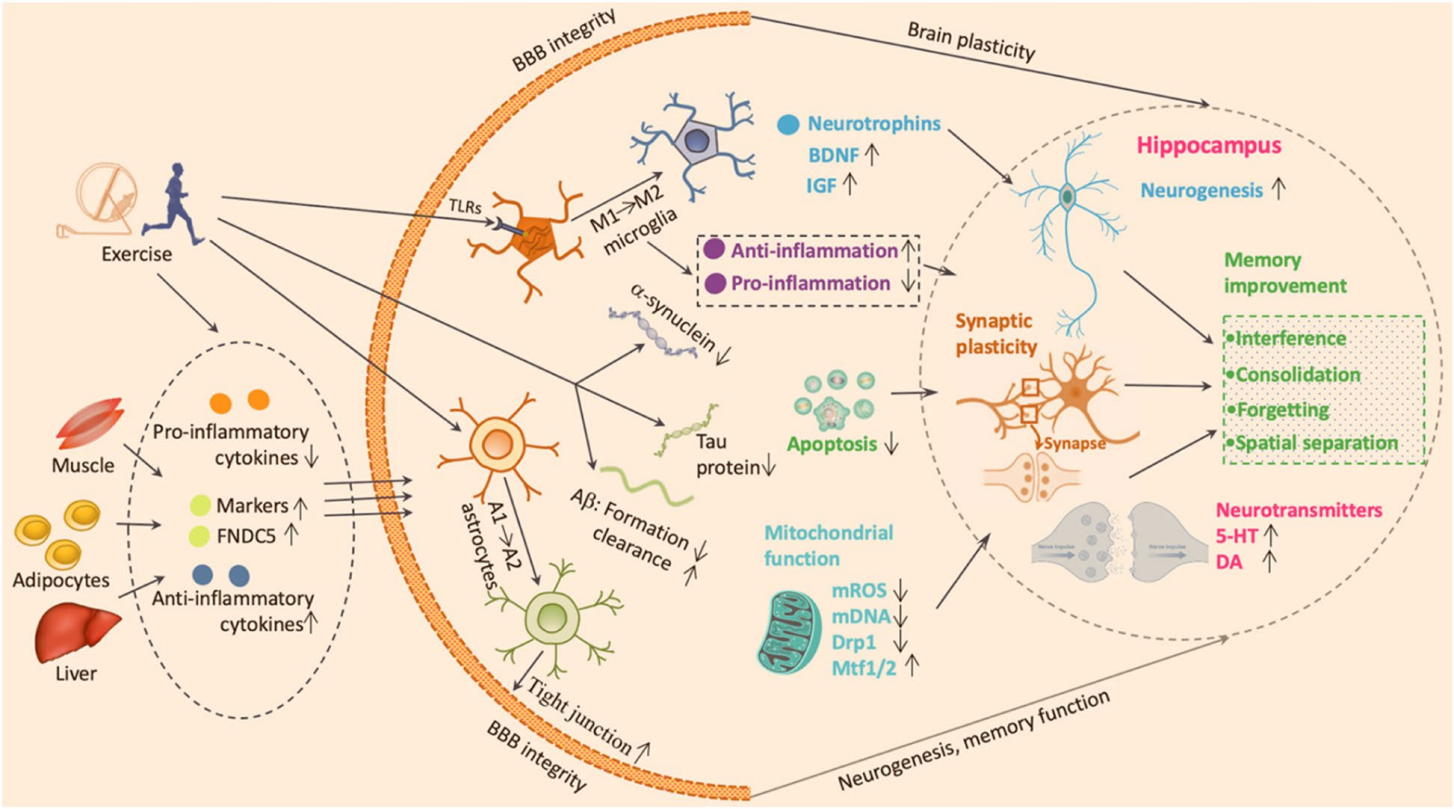
Exercise for hippocampal neurogenesis in neurodegenerative conditions. Physical exercise can exert multiple positive effects on AD and PD brains, such as enhancing cerebral blood flow, neurogenesis, synaptic plasticity, neurotrophic factors, antioxidant defense, and cognitive function. Exercise can inhibit Aβ formation and deposition, abnormal phosphorylation of Tau and α-synuclein aggregates. More importantly, physical exercise can increase hippocampal neurogenesis and memory function by modulating mitochondrial dysfunction, neuronal apoptosis, and neuroinflammation. Physical exercise can impact the activation state and phenotype of microglia and astrocytes in AD, resulting in the shift of the polarization of microglia and astrocytes from pro-inflammatory (M1 or A1) to anti-inflammatory (M2 or A2) pattern. Additionally, physical exercise can strengthen the connection of BBB, which can prevent the infiltration of peripheral immune cells and inflammatory molecules into the brain. AD, Alzheimer's disease; PD, Parkinson’s disease; Aβ, Amyloid beta; BBB, the blood–brain barrier; BDNF, Brain-derived neurotrophic factor; IGF, Insulin-like growth factor; Drp1, Dynamin-related protein 1; Mtf1/2, Mitofusion protein 1; 5-HT, 5-hydroxytryptamine; DA, Dopamine; FNDC5, fibronectin type III domain-containing protein 5; mROS, Mitochondrial reactive oxygen species

Exercise can yield various benefits for brain function, such as increasing brain volume, cerebral perfusion, hippocampal functions, and synaptic connectivity in healthy adults and patients with neurodegenerative disorders [111–113] (Fig. 2). These beneficial effects are partly involved in the up-regulation of biomarker production. Exercise can increase the levels of various biomarkers that are involved in adult neurogenesis, in peripheral tissues and cerebrospinal fluid (CSF) [114–116], such as BDNF, insulin-like growth factor (IGF)-1, irisin, vascular endothelial growth factor (VEGF), fibroblast growth factor (FGF)-2, glial cell line-derived neurotrophic factor (GDNF), and DCX. Furthermore, physical exercise also influences the levels and activities of various neurotransmitters and neuropeptides in the human brain (Fig. 2), such as glutamate, GABA, serotonin (5-HT), dopamine (DA), norepinephrine, and acetylcholine (ACh). These neurotransmitters play crucial roles in regulating adult neurogenesis by influencing neural stem cell proliferation, differentiation, migration, and integration [117, 118]. Additionally, exercise has been shown to decrease the levels of pro-inflammatory cytokines and improve the levels of anti-inflammatory cytokines in the peripheral blood or CSF of humans [34, 119, 120], which can be advantageous for adult neurogenesis by modulating neuroinflammation and neuroimmune interactions. Together, growing evidence suggests that physical exercise can improve brain blood flow, oxygen delivery, glucose metabolism, and neurotrophic factors within the brain, as well as regulating inflammation, oxidative stress, mitochondrial function, and epigenetic modification. These processes are governed by various molecular and cellular signalling pathways, but the precise mechanisms remain unclear.

### Exercise restores adult neurogenesis in neurodegenerative disorders

One of the mechanisms by which exercise alleviates neurodegeneration and improves cognition is through enhancing AHN [10, 121] (Fig. 2). Growing evidence from in vivo and in vitro experiments has demonstrated that physical exercise can protect brain plasticity and function in ageing rodents and those with neurological conditions. These changes include promotion of the number, the survival, the differentiation, and the integration of newly generated neurons in the brain, as well as the increase of their synaptic connections [10, 122, 123] (Fig. 2). Recent studies have found that different types of exercise training, such as wheel running, treadmill running, and swimming, can promote the proliferation, survival, differentiation, and integration of new neurons in the DG region of the adult rodent brain [124–127]. Moreover, exercise can also improve synaptic plasticity and the function of these new neurons, resulting in more complex dendrites, more spines, stronger LTP, and higher electrophysiological activity [102, 6, 112]. Accordingly, rodents that received exercise showed improved cognition and mood in various hippocampal-dependent tasks, such as spatial learning, memory consolidation, pattern separation (PS), and depression-like behaviour [128–130].

The regulatory mechanisms by which exercise promotes adult neurogenesis in rodents are complex and multifactorial, involving neurotrophic factors, neurotransmitters, inflammation, oxidative stress, epigenetics, and mitochondrial functions [9, 34, 114, 116, 131–133] (Fig. 2). Exercise can increase the expression and secretion of various growth factors, such as BDNF, irisin, IGF-1, VEGF, FGF-2, and GDNF, which can support the survival, growth, and differentiation of new neurons [114–116, 134]. Moreover, the levels and activities of neurotransmitters that influence adult neurogenesis are also elevated in rodents after exercise, such as GABA, 5-HT, DA, NE, ACh, endocannabinoids, and neuropeptides [117, 118]. Additionally, recent studies have found that exercise can reduce the production of pro-inflammatory cytokines and increase the production of anti-inflammatory cytokines (IL-4, IL-10, IL-1 receptor antagonist, etc.) in the brain, which can have beneficial effects on adult neurogenesis by modulating microglial activation, astrocyte function, blood–brain barrier (BBB) permeability, and immune cell infiltration [34, 120, 135, 136]. Similarly, exercise has been shown to enhance the antioxidant defence system in the brain by increasing the expression and activity of antioxidant enzymes, such as superoxide dismutase, catalase, glutathione peroxidase, and glutathione reductase (GR) [137–141]. Exercise can also reduce brain oxidative damage to DNA and proteins [142], and alter epigenetic regulation of gene expression by modulating DNA methylation, histone modifications, and non-coding RNAs [143–147]. Together, physical exercise is capable of producing various neurobiological advantages that promote brain health and counteract neurodegeneration in both ageing individuals and those with neurodegenerative diseases. These findings indicate that exercise could be a promising strategy for managing brain disorders.

## Exercise mimetics: a pill for neurogenesis and neurological disorders

### Exercise mimetic is a new strategy for neurological disorders by boosting neurogenesis

There is an increasing consensus on the positive effects of physical exercise in a broad spectrum of models of human diseases [148, 149], particularly CNS disorders such as AD and PD [150]. Besides the therapeutic effects on the pathological process of diseases, exercise also has a favourable impact on cognitive function in neurodegenerative disorders [6, 8, 33, 151, 152]. It is fairly clear, as many studies have discussed [38, 153, 154], that exercise could be developed as a pharmacological strategy for neurodegeneration and cognitive dysfunction, presenting as exercise mimetics [38, 39, 155] (Table 1). This becomes particularly important for older adults and patients with injuries or severe neurological diseases who have difficulties in conducting exercise regimens. Exercise mimetics are an attempt to let ‘exercise effects’ become available to the whole population through pharmacological proteins or factors (such as BDNF, Clusterin, irisin, and IGF-1) (Table 1). Interestingly, several featured studies have been conducted recently to explore the potential of ‘exercise mimetics’ in improving brain functions in neuropathological or ageing conditions [34, 36, 37]. Understanding the molecular and cellular processes underlying the communications between the periphery and the brain (such as muscle-brain crosstalk, liver-brain axis, and gut-brain axis) in response to exercise can facilitate development of exercise mimetics. We here particularly focus on the molecular effects of exercise mimetics on adult neurogenesis and cognitive function in neurological disorders.

**Table 1:** Candidate molecules of exercise mimetics for the treatment of neurodegenerative disorders

Type of exercise mimetics	Molecule	Compound	Beneficial effects	Therapeutic implications	Key studies
Myokines	Irisin	Recombinant irisin	(1) Attenuate glial activation by binding αVβ5 integrin receptor; improve spatial learning and memory (2) Decrease tau phosphorylation, α-synuclein, and inflammatory cytokine levels (3) Rescue synaptic plasticity and improve impairment in NOR and fear conditioning memory	Anti-neuroinflammation, neuronal protection and memory promotion for AD and PD	[181, 190–192]
	AMPK	Metformin	(1) Improve cognitive function (2) Reduce the risk of dementia	AD prevention and cognitive promotion for AD and PD	[337–339, 354]
		AICAR	(1) Increase IDE and reduce Aβ or α-synuclein deposition; enhance spatial learning and cognitive performance (2) Inhibit neuroinflammation and promote neurite growth	Neuroinflammation inhibition, neuronal protection and cognitive improvement for AD and PD	[330–332, 355, 356]
	PPARδ	GW501516	(1) Increase AHN and cognition (2) Reduce neuroinflammation	Brain health and cognitive promotion for AD	[347, 348]
Neurotrophic factor	BDNF	Recombinant BDNF, Recombinant BDNF + ADTC5, DHF	(1) Up-regulate α-secretase, ameliorate Aβ levels and increase sAPPα levels (2) Reduce α-synuclein level (3) Increase phosphorylation of TrkB and restore synaptic plasticity and memory function (4) Increase AHN and memory performance (5) Increase NG2 glial cells and EGR1 and ARC gene expression; enhance cognitive performance and NOR assessments	Neuronal protection and cognitive improvement for AD, PD, and HD	[36, 155, 167, 357–359]
Noncoding RNAs	miRNAs	miRNA-132, miRNA-146, miRNA-155, miRNA-7, miRNA-21, miRNA-339, miRNA-29, miRNA-129–1-3p, miRNA-144-5p, miRNA-708-5p, miRNA-135a, miRNA-21, miRNA-34a	(1) Rescue AHN and memory impairment (2) Alleviate the accumulation of α-synuclein or Aβ and Tau hyperphosphorylation; restore AHN and cognitive function (3) Ameliorate neuroinflammation (4) Ameliorate the activation of microglial cells and neurodegeneration	Anti-neuroinflammation; brain and memory improvement for AD, PD and HD	[147, 272, 276, 277, 284, 286, 300, 306, 360–366]
Methyl donor	Methylation	SAM	(1) Ameliorate AD pathology and improve cognitive function (2) Restore normal gene expression, reduce presenilin1 expression, and decrease Aβ levels。 (3) Increase secretases and ApoE expression (4) Increase GSH and decrease ROS and toxic xenobiotics (5) Prevent neuronal loss, elevate BDNF levels, and inhibit neuroinflammation	Brain health and memory promotion for AD and PD	[250–253, 255, 257, 367]
Metabolism mediators	Apolipoprotein J	Clusterin	(1) Inhibit neuroinflammation, promote AHN, and improve contextual learning and memory function (2) Increase excitatory neurotransmission, reverse synaptic damage and alleviate AD pathology (3) Reduce cell apoptosis and increase neuronal differentiation	Hippocampal neurogenesis and memory improvement for AD	[34, 315, 368]
	GPI	Gpld1	Increase AHN, BDNF level, and memory function	Brain and memory promotion for AD	[37]
Other candidates	Gut microbiome	SCFAs	(1) Increase gut health and preserve microbiota community diversity (2) Enrich SCFAs, Firmicutes phylum (3) Decrease gut inflammation and improve cognition	Gut-brain health and cognitive improvement for AD, PD and HD	[224, 227, 369–375]
	VEGF	SU1498	Promote AHN and cognition	Brain health and memory promotion	[376–378]
	Serotonin	TPH2, 5-HT3 receptor, SERT	Increase neuronal proliferation and outgrowth	Brain health promotion	[324, 379–381]

*BDNF* Brain-Derived Neurotrophic Factor, *Aβ* Amyloid Beta, *sAPPα* Soluble Amyloid Precursor Protein Alpha, *TrkB* Tropomyosin Receptor Kinase B, *AHN* Adult Hippocampal Neurogenesis, *EGR1* Early Growth Response Protein 1, *ARC* Activity-Regulated Cytoskeleton-associated Protein, *NOR* novel object recognition, *ADTC5* Alzheimer's Disease Transmembrane Conductance Regulator 5, *DHF* Dihydrofolate, *AD* Alzheimer's Disease, *PD* Parkinson’s diseases, *HD* Huntington’s disease, *AMPK* AMP-Activated Protein Kinase, *AICAR* 5-Aminoimidazole-4-carboxamide Ribonucleotide, *PPARδ* Peroxisome Proliferator-Activated Receptor Delta, *IDE* Insulin-Degrading Enzyme, *miRNA* MicroRNA, *SAM* S-Adenosyl Methionine, *GSH* Glutathione, *ROS* Reactive Oxygen Species, *GPI* Glycosylphosphatidylinositol, *Gpld1* Glycosylphosphatidylinositol-Specific Phospholipase D1, *SCFAs* Short-Chain Fatty Acids, *VEGF* Vascular Endothelial Growth Factor, *TPH2* Tryptophan Hydroxylase 2, *SERT* Serotonin Transporter, *5-HT3* 5-Hydroxytryptamine Receptor 3

#### BDNF

**Fig. 3:**
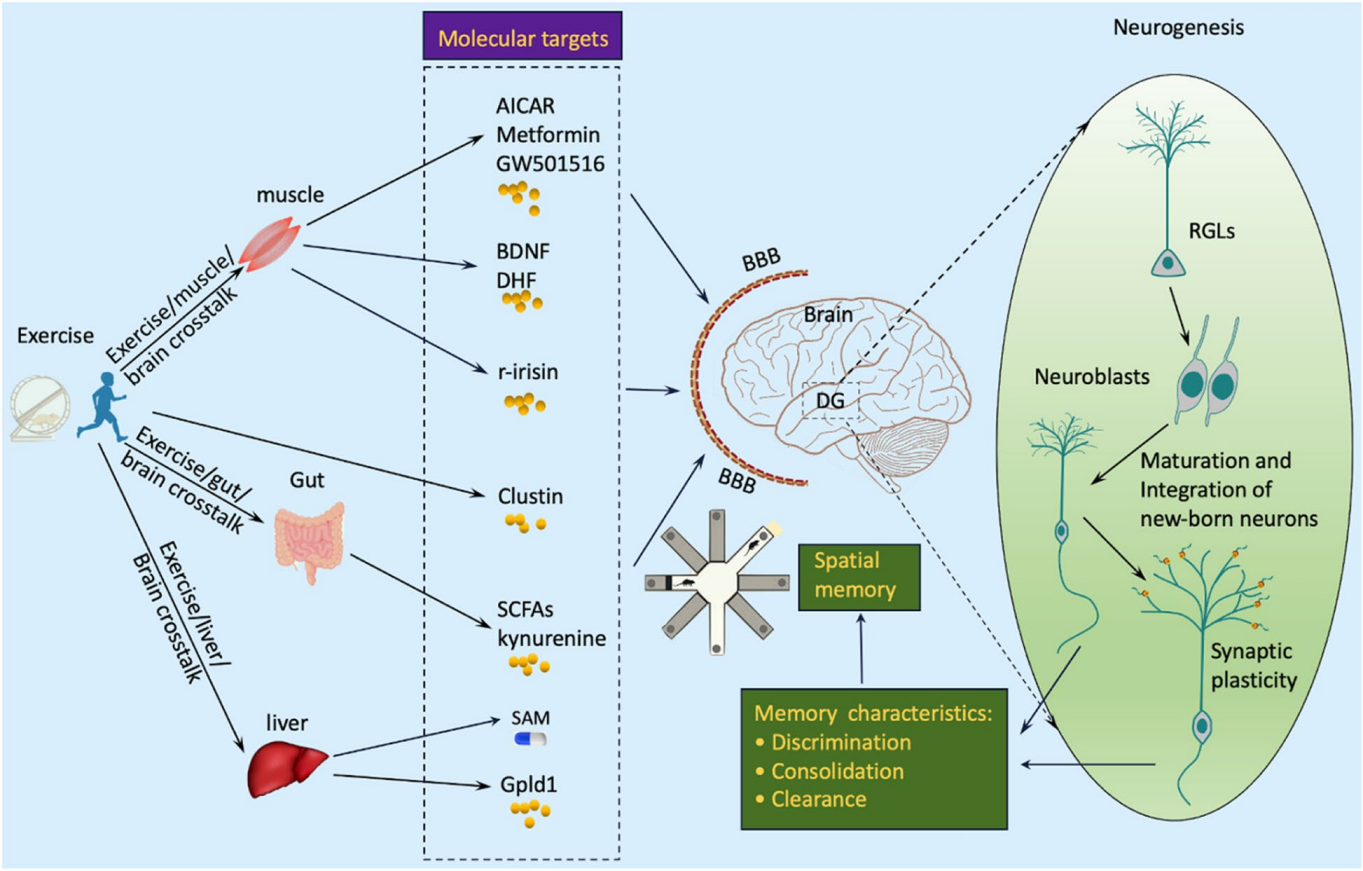
Impact of exercise on brain plasticity and function through intercommunications between muscle, gut, liver, and brain. During exercise, a wide range of molecules, factors, or cytokines are secreted from different organs or tissues, such as muscle, liver, and intestinal tract, and enter blood flow, directly or indirectly affecting the central nervous system (CNS). It is fairly clear that exercise could be developed as a pharmacological strategy for alleviating neurodegeneration and cognitive dysfunction, presenting as exercise mimetics [38, 39, 155]. MARK, Adenosine monophosphate-activated protein kinase; SIRT1, Sirtuin 1; PGC-1α, Peroxisome proliferator-activated receptor coactivator 1α; FNDC5, Fibronectin type III domain-containing protein 5; Gpld1, Glycosylphosphatidylinositol (GPI)–specific phospholipase D1; BDNF, Brain-derived neurotrophic factor; DHF, 7, 8-dihydroxyflavone; SCFAs, Short-chain fatty acids; RGLs, Radial glia-like stem cells

BDNF is a member of the neurotrophin family and is highly expressed in the brain. Peripherally secreted BDNF can cross the BBB and reach the brain [156, 157]. The levels of BDNF can be affected by various neurodegenerative disorders, including PD, AD, and HD. Patients with these conditions often have reduced circulating levels of BDNF compared to matched healthy controls [158–160]. BDNF is one of the most intensively investigated exercise mimetics due to its close relation to physical exercise and crucial role in mediating neuronal proliferation, maturation, survival, and integration into existing circuits [161–163] (Fig. 3). The exercise-induced increase of adult neurogenesis and improvement of brain function are proposed to be associated with up-regulation of BDNF levels in the hippocampus [9, 164, 165]. BDNF exerts neurobiological impact primarily through its receptor, tropomyosin receptor kinase B (TrkB) [166]. Interestingly, exercise interventions effectively protect against neuropathology and cognitive dysfunction and increase BDNF expression in animal models of AD [24], HD [167], and other neurodegenerative disorders [163]. Moreover, BDNF^Met/Met^ mutant mice display impairments in BDNF expression, hippocampal neurogenesis, and behavioural performance compared to BDNF^Val/Val^ wild-type mice, which could not be reversed by exercise intervention, indicating that the presence of BDNF is essential for the neurobiological effects of exercise [168]. According to those data, pharmacologically manipulating hippocampal BDNF expression may replicate in part the neurobiological effects of exercise. Parrini and colleagues [155] increased hippocampal BDNF levels in Ts65Dn mice (a transgenic model of Down syndrome) via chronic administration (5 mg/kg body weight, 4 weeks) of 7, 8-dihydroxyflavone (DHF). Chronic DHF intervention, in contrast to acute treatment [169], directly induced a 26% increase in phosphorylated TrkB levels and successfully restored hippocampal synaptic plasticity and cognitive function [155].

Recently, one prominent study conducted by Choi and colleagues [36] combined BDNF protein and drug-induced neurogenesis to recapitulate the effect of exercise on cognitive performance in a transgenic rodent model of AD. They reported that only the combined protocols, instead of either alone, produced the exercise-related neurobiological effects (particularly cognitive function), demonstrating that the application of exercise mimetics in certain animal models is more complicated than expected. Additionally, apart from BDNF, exercise also increases expression of several other proteins, including postsynaptic density protein 95, synaptophysin, IL-6, and fibronectin type III domain containing 5 (FNDC5), some of which have been recognized to be associated with the neuropathology of AD [170, 171]. Given the critical role of BDNF in the effects of exercise on cognitive function, anxiety, and depression-like behaviours via mediating neurogenesis and synaptic plasticity [172–174], BDNF has the potential to be a promising molecular target of exercise mimetics.

#### FNDC5/Irisin

Exercise improves brain function partly through production of myokines from muscles to target the CNS, such as irisin. Irisin is a novel myokine initially identified in muscles and adipose, consequently extending to the heart, the liver, the bone, and the brain. It generates the new molecular basis for the crosstalk between muscles and the brain [134, 175, 176]. Irisin secretion is stimulated by exercise and regulated by peroxisome proliferator-activated receptor-γ (PPARγ) coactivator 1α (PGC-1α), a transcriptional co-regulator involved in energy metabolism [177]. Compelling studies involving individuals with AD and PD have implied a close relationship between circulating irisin levels and the risk of developing AD and PD [178–183]. Moreover, the preventive effects of irisin treatment against neuropathology have been extensively investigated in various rodent models of neurological disorders [181, 184–189]. Bretland et al., [190] showed that a 4-week injection of recombinant irisin (100 µg/kg, weekly) significantly decreased the tau phosphorylation load and inflammatory cytokine levels such as TNF-α in the hippocampus of female htau mice. Similarly, the recombinant irisin also improves novel object recognition and fear conditioning memory in AD mice, and over-expression of FNDC5/irisin rescues synaptic plasticity and memory defects in AD mice [191]. A recent study on rodent PD models showed that irisin intervention improves motor function and prevents dopaminergic neurodegeneration by reducing oxidative stress, restoring mitochondrial function, and normalizing mitochondrial dynamics and morphology [181]. Moreover, the presence of irisin is indispensable for exercise-associated improvement of brain plasticity and function. Islam and colleagues generated *Fndc5* KO mice and housed each in a cage with free access to a voluntary running wheel. *Fndc5* KO mice did not show exercise-related beneficial effects in spatial learning and memory compared with wild-type mice [192]. Moreover, while *Fndc5* KO rodents displayed difficulty in PS tasks, overexpression of irisin by injecting adeno-associated virus (AAV) 8-irisin-FLAG in hippocampal DG restored PS in *Fndc5* mutant mice [192]. To further test the therapeutic potential of irisin, several studies have been conducted by peripherally overexpressing or blocking irisin to test its impact on cognitive function [191, 192]. Lourenco et al*.* reported that swimming exercise (1 h per day, 5 days per week for 5 weeks) improved memory and hippocampal *Fndc5* expression in mice with Aβ oligomer infusion [191]. Interestingly, the effects of exercise could be recapitulated, to some extent, by peripherally overexpressing FNDC5/irisin, which resulted in increased hippocampal irisin level and reversed AD-related memory deficits and neural pathology [191]. Additionally, the effects of exercise on synaptic plasticity and memory of AD mice could be inhibited by peripherally or cerebrally blocking FNDC5/irisin [191]. Those findings suggest that irisin could recapitulate partial ‘exercise effects’, indicating that it is a potential molecular exercise mimetic to improving brain plasticity and function (Fig. 3).

Irisin is implicated in exercise-related cerebral plasticity and function probably via modulating the process of neurogenesis through up-regulating hippocampal BDNF expression. Exercise cannot rescue the impairment of the morphology and maturation of newborn neurons in the hippocampus of *Fndc5* KO mice [192]. Additionally, *Fndc5* KO during differentiation of rodent embryonic stem cells into neurons resulted in remarkable down-regulation of neural progenitor markers, such as *Sox1, Sox3, paired box 6,* and *Nestin* [193]. It is hinted that the presence of irisin is critical for exercise-induced beneficial effects on neuronal proliferation and maturation. The capacity of irisin to improve brain plasticity and function is partly through increasing BDNF expression in the brain. Wrann et al*.* [194] demonstrated that forced expression of *Fndc5* remarkably increased *Bdnf* expression in primary cortical neurons, whereas *Fndc5* deletion via lentiviral delivery of shRNA markedly decreased *Bdnf* expression. Interestingly, peripheral overexpression of *Fndc5* in the liver resulted in elevated serum level of irisin and enhanced hippocampal *Bdnf* expression in mice [194]. Recently, Choi et al*.* [36] demonstrated that exercise remarkably increased adult neurogenesis in transgenic AD mouse models together with elevated levels of hippocampal FNDC5 and BDNF. Recent evidence proposes an important role of irisin in mediating muscle-brain communications in exercise via a PGC-1α/irisin/BDNF-dependent pathway [134, 195]. Given the close relationship between irisin and BDNF, pharmacological or exercise-induced expression of irisin is likely to increase cerebral BDNF levels and consequently favor hippocampal neurogenesis and cognition in various brain conditions [9, 194, 196].

#### Gut microbiota

The microbiota-gut-brain axis is an emerging field of interest and a promising therapeutic target for CNS disorders [197]. Changes in gut microbial profiles have been reported in diverse brain disorders, including PD [198–201], AD [202, 203], and major depressive disorder [204, 205]. More interestingly, transplantation of faecal microbiota from patients with PD [206] or depression [207] led to phenotypes of disease in the recipient mice. On the contrary, transplantation of the intestinal microbiota from wild-type mice to ADLP^APT^ mice (a transgenic mouse model of AD) alleviated Aβ plaque deposition, neurofibrillary tangle formation, and cognitive deficits [208]. Growing evidence has suggested that changes in intestinal microbiota can potentially affect AHN [209–211], neuroplasticity [212], and cognition [213, 214] in neurodegenerative diseases and in ageing. In a recent study, Kim et al*.* [211] transferred fecal microbiota from 5 × FAD transgenic mice to normal C57BL/6 mice, which resulted in reduced AHN and BDNF expression, increased p21 expression, and memory impairment. The gut microbiota composition of the 5 × FAD mice differed from that of the control or wild-type mice, accompanied by elevated microglial activation in the hippocampus and increased pro-inflammatory cytokines in both the colon and the plasma [211]. Another study transplanted fecal microbiota from AD patients into microbiota-depleted young adult rats, which resulted in impairments in AHN-related behaviours in the recipient rats [209]. Moreover, human neural cell culture showed reduced neurogenesis when exposed to serum from AD patients. Importantly, the extent of neurogenesis reduction correlated with the cognitive scores of the AD patients, highlighting the significant influence of gut microbiota on hippocampal neurogenesis in AD [209]. These data suggest that altering the gut microbiota, possibly through exercise or products targeting its enrichment, could offer a new strategy for enhancing AHN and cognitive function in neurodegenerative conditions [215].

There is increasing evidence that physical exercise benefits both the body and the brain. One mechanism is through modification of gut microbiota, including decrease in gut transit time that leads to reduced colonization of pathogenic bacteria in the gut, and increases in diverse microbiota profiles, production of short-chain fatty acids (SCFAs), and gut microbiome composition [216–221]. In an American Gut Project, gut microbiota from individuals with regular exercise (3–5 times per week) showed a greater diversity, with enrichment in certain members of the Firmicutes phylum, such as *Faecalibacterium prausnitzii, Lachnospira, Oscillospira,* and *Coprococcus* [222]. The gut microbiota has been proposed to account for the effects of exercise on neurodegenerative disorders via enhancing AHN and cognitive function. Accordingly, exercise intervention dramatically increases the microbial enrichment in high-fat diet mice, leading to cognitive improvement [223]. In a mouse model of AD, exercise significantly ameliorated cognitive dysfunction and neuropathological biomarkers of AD, companied by microbiome alterations favouring SCFA-producing bacteria [224]. As mentioned previously, the enrichment of gut microbiota is important for AHN. The changes in gut microbiota in response to exercise may therefore benefit hippocampal neurogenesis and cognition. Indeed, research has shown that sedentary rats with disrupted microbiota display impaired performance in hippocampal neurogenesis-dependent tasks, such as the modified spontaneous location recognition task and the novelty-suppressed feeding test [225]. However, voluntary exercise is able to alleviate these effects and leads to increased hippocampal neurogenesis, which is linked to changes in caecal metabolomics [225]. Moreover, Mohle et al*.* [210] found that exercise training led to a moderate improvement of AHN in antibiotic-treated mice. Interestingly, when the antibiotic-treated mice received a fecal transplant from wild-type murine, the exercise-induced neurogenesis was increased by 47%, equivalent to the effect observed in control mice. This highlights the essential role of gut microbiota in mediating the effects of exercise on AHN. Therefore, the potential of physical exercise in increasing AHN by modulating the gut microbiome offers a promising protocol for the treatment of cognitive deficits associated with ageing and neurodegenerative diseases [225, 226].

Although the exact regulators driving such beneficial effects remain largely unclear, SCFAs, as the major microbial metabolites of dietary fibre in the gut, are speculated as an essential mediator. Evidence from animal models suggests that SCFAs might directly cross the BBB and affect brain function via their receptors, free fatty acid receptors [227]. Additionally, SCFAs can also interact with diverse immune cells to modulate systematic inflammation and control microglial integrity and activation that are involved in neuroinflammation [227]. PD patients often have a reduced enrichment in SCFA-producing bacteria and lower faecal SCFA contents compared with healthy controls [198, 200, 228]. Interestingly, butyrate administration ameliorated motor deficits and dopamine deficiency in rodent PD models [229, 230] and restored cognitive performance and expression of transcripts involving memory function in an AD mouse model [231]. To investigate the effects of SCFAs on neurogenesis, Xiong et al*.* [232] performed a single-cell mRNA analysis on samples from mice with traumatic brain injury. The results showed that SCFAs increased the frequency of immature neurons and downregulated the expression of genes associated with neurodegenerative diseases during the differentiation of immature neurons in the injured mice. Moreover, SCFAs can rescue hippocampal neurogenesis, improve BBB damage, and suppress microglial activation and neuroinflammation in mice fed a high-fructose diet and exposed to chronic stress, ultimately alleviating depressive-like behaviours [233]. The beneficial effects of SCFAs on neurogenesis, BBB integrity, and neuroinflammation suggest that these metabolites may have therapeutic potential for treating cognitive deficits and other neurological disorders.

Another mediator is kynurenine, which is strongly associated with depression. Kynurenine is proposed to be involved in the central effects of gut microbiota, which is linked with the capacity of gut microbiota to control host tryptophan metabolism and circulating kynurenine levels [234]. Physical activity increases PGC-1α expression in muscles and consequently up-regulates the kynurenine signalling cascade in the brain [235], which consequently results in resilience to stress-induced depression and CNS inflammation [235]. Taken together, the management of gut microbiota is a promising approach to recapitulating the beneficial effects of exercise on brain function (Fig. 3). One unique advantage of this strategy is the availability of a range of therapeutic products, such as probiotics and prebiotics (the applications of those products have been reviewed elsewhere [197, 227, 236]). In a recent study, Mohle et al. conducted an interesting experiment that may provide insights into the relationship between gut microbiome management by probiotics and AHN [210]. They found that simply normalizing the overall species distribution of the gut flora in mice with antibiotic-induced impairment of AHN had limited effect in restoring hippocampal neurogenesis. However, when the antibiotic-treated mice received a probiotic intervention, the hippocampal neurogenesis was completely restored to control levels [210]. This suggests that introducing beneficial probiotic bacteria may be an effective approach to restoring hippocampal neurogenesis. However, questions remain to be resolved in this field. For example, which particular microbial species are mostly affected by exercise in relation to cerebral function? Understanding this helps to discern novel candidate targets of exercise mimetics. In addition, identification of relevant mediators and elucidation of how those factors work in microbiota-gut-brain communications in response to exercise remain to be addressed in future studies.

#### S-Adenosyl methionine (SAM)

SAM is known as a methyl donor that provides a methyl group to another molecule through the process of methylation [237, 238]. Methyl donors are involved in diverse biological processes, including DNA methylation. DNA methylation has the potential to impact gene expression, protein synthesis, neurogenesis, and neurotransmitter metabolism [239], which are frequently disrupted in neurological conditions like AD and PD [240, 241]. 5-Methylcytosine (5mC) is an important epigenetic pattern of DNA methylation. It functions through the recognition by the methyl-binding domain (MBD) 1, which serves as an essential partner in the process of adult neurogenesis [242]. In vitro, cultured MBD1-deficient NSCs exhibit reduced neuronal differentiation and increased genomic instability [243]. Furthermore, adult mice lacking MBD1 show impaired neurogenesis and spatial learning abilities [243]. Additionally, another study found that the stimulation of mature dentate neurons in the hippocampus in adult mice via electroconvulsive therapy resulted in a prolonged elevation of hippocampal neurogenesis [244, 245]. This phenomenon is linked to the upregulation of the *Gadd45b* (growth arrest and DNA-damage-inducible protein 45) gene in the brains of these mice. Gadd45b plays a pivotal role in the demethylation of particular gene promoters and the activation of genes that are fundamental to the process of neurogenesis in adults, such as *BDNF* and *FGF-1* [244, 245]. Growing evidence has revealed significant alterations in the methylation profiles of specific genes in AD patients, indicating the potential involvement of methylation in AD pathogenesis. A recent meta-analysis of methylation data in various brain regions of AD highlighted the enrichment of methylation changes in genes crucial for neurodevelopment and neurogenesis [241]. DNA methylation is under dynamic regulation by various factors, including DNA repair, oxidative stress, inflammation, environmental stimuli, and exercise [246], which may subsequently influence brain functions such as neurogenesis, synaptic plasticity, and cognition. These hint at potential therapeutic implications of DNA methylation in regulating the development and progression of AD pathology [246].

Exercise is a dynamic mediator for methylation patterns in brain health and disorders [247]. For instance, among a cohort of elderly African Americans with mild cognitive impairment, a 6-month regimen of aerobic exercise (40 min per day, 3 days per week) led to global DNA methylation changes [143], including methylation of *VSP52*, *SACRB1*, *ARTN*, *NR1H2*, and *PPPLR5D*, which play roles in diverse processes such as amyloid formation, intracellular protein transport, and lipoprotein mediation [143]. Additionally, exercise alters the activity of DNA methyltransferases, which are involved in the regulation of neuronal survival and methylation processes associated with ageing and disease-related neurodegeneration [248]. Swimming for 1 h a day, 26 days, increased the protein level and the DNA-binding activity of Nrf2 (nuclear factor erythroid 2-related factor 2) in a rodent model of AD, which subsequently led to decreased expression of antioxidant genes [249]. Considering the important role of gene methylation in the beneficial effects of exercise on brain function, DNA methylation and associated signalling pathways, including methyl donors, are potential therapeutic targets for brain disorders like AD and PD.

As an essential methyl donor, SAM can be used as both a dietary supplement and a prescription drug for various conditions, including depression, anxiety, liver disease, osteoarthritis, PD, and AD. Di Rocco et al. [250] treated PD patients with SAM at doses of 800 to 3600 mg/day for 10 weeks and observed a significant improvement on the 17-point Hamilton Depression Scale. Furthermore, SAM improved AD pathology and cognitive function. In 3 × Tg-AD mice, SAM administration (100 mg/kg) reduced intracellular Aβ deposits and phosphorylated tau in the hippocampus [251]. SAM therapy decreased AD neuropathology in a time-dependent manner, showing an 80% decrease in extracellular Aβ deposition in 11-month-old mice following 1-month treatment, but only a 24% reduction in 15.5-month-old mice after 3-month treatment [251]. SAM potentially influences Aβ metabolism by controlling its production, removal, and clumping. As a methyl donor, SAM can affect the functions of enzymes like secretases, which are involved in preventing generation or breakdown of APP [252]. Additionally, SAM may boost Aβ elimination by raising the levels of ApoE [253] and hinder Aβ clumping by interrupting its interaction with metal ions or changing its structure [254].

SAM treatment can also regulate certain disease processes such as oxidative damage, inflammation, mitochondrial dysfunction, and cholinergic deficits. For instance, SAM exerts antioxidant properties by increasing glutathione (GSH) and its transferase, which are crucial for neutralizing oxidative substances and removing harmful foreign substances. SAM supplementation increases glutathione S-transferase activity and the removal of reactive oxygen species (ROS) in ApoE^−/−^ mice [255, 256]. Furthermore, in rats with *D*-galactose-induced brain ageing, SAM treatment (16 mg/kg for 4 weeks) reverses cognitive decline, rescues neuron loss, increases BDNF levels in the hippocampus, suppresses microglial activation, and decreases the levels of pro-inflammatory cytokines in the hippocampus and serum [257]. Additionally, SAM (10 mg/kg daily for 6 weeks) attenuated Aβ-induced cellular damage by suppressing neuroinflammation and oxidative stress [258]. Mitochondrial impairment is an essential feature of brain disorders [259] and represents a promising treatment target. Recently, Lam et al. [260] found that addition of vitamin B12 in the diet can alleviate mitochondrial fragmentation, bioenergetic defects, and oxidative stress, delaying Aβ-induced paralysis in a methionine/SAM-dependent manner. The attenuation of neuropathological conditions, such as neuroinflammation, oxidative stress, and mitochondrial dysfunction, as well as the increased expression of BDNF and other genes that regulate the process of hippocampal neurogenesis, is proposed to enhance hippocampal neurogenesis and cognition [36, 261–263]. In folate-deficient mice, intraperitoneal injection of SAM at a dose of 50 mg/kg per day for 14 days, starting at the age of 7 weeks, prevented an excessive number of immature neurons (DCX^+^ cells) while simultaneously promoting the maturation of new neurons in the DG [264]. Furthermore, SAM supplementation improves dendritic complexity and increases the number of mature spines in the DG [264]. Proper intake of methyl-donor nutrients is important for brain development and cognition. Decreased methyl-donor nutrients in the developing fetus can adversely affect brain development, significantly increasing offspring risk for metabolic and neurological disease development [265]. For example, mice fed a low folate diet show depression-like behaviour [266]. These mice display neuronal immaturities, such as increased immature neurons, decreased newborn mature neurons, reduced dendrite complexity and mature dendritic spines in the dentate gyrus (DG), accompanied by down-regulation of transcription factors involved in neuronal differentiation and maturation in the DG [266, 267]. In contrast, as demonstrated in a study by Wang [268], supplementation of the methyl donor folic acid (4–12 mg/kg, 3 times per week, for 4 weeks) improved cognition and restored the expression of BDNF in the hippocampus of mice with inhibited DNA methylation. Moreover, this treatment also improved hippocampal neurons and increased expression of pro-neurogenic genes, including DNA methyltransferase 1, histone deacetylase (HDAC) 6, and HDAC8 [268]. Collectively, SAM and other methyl-donors have demonstrated beneficial effects on neuropathological conditions associated with neurodegenerative diseases [269]. However, SAM treatment may face some limitations, including the absence of a clearly defined optimal dosage, duration, and method of administration, as well as the potential side effects. Additionally, there is a lack of well-designed, population-based studies evaluating the impact of SAM administration on hippocampal neurogenesis and cognition in various neurodegenerative diseases. Therefore, future studies are needed to address these issues.

#### MicroRNAs (miRNAs)

MiRNAs are short non-coding RNAs that bind mRNAs and affect their translation or degradation. MiRNAs affect diverse physiological and pathological aspects of brain health and diseases such as AD, by controlling the expression of genes related to Aβ formation, tau phosphorylation, neuroinflammation, synaptic plasticity, and neuronal viability [270–273]. Growing evidence has indicated a close relationship between miRNAs and neurological diseases [270, 271, 274–276]. Recently, Walgrave et al. [277] have suggested that in AD, the expression of miR-132 is down-regulated by Aβ deposition. The decrease of miR-132 is associated with impaired AHN in the DG of the hippocampus in transgenic AD mice. In addition, restoring miR-132 level in adult AD mouse hippocampus rescues AHN and memory deficits, suggesting that miR-132 is a potential biomarker and therapeutic target for brain disorders. Besides regulating gene expression in brain disorders, miRNAs are also key signalling molecules involved in brain response to physical exercise by forming regulatory complexes that influence various neurobiological processes [278–282]. For example, the levels of miR-106a-5p, miR-103a-3p and miR-29a-3p are significantly elevated after an 8-week exercise intervention that improves cognitive performance in PD patients [283]. In a similar study, voluntary physical exercise ameliorated the degradation of the pro-neurogenic environment in the hippocampus of a mouse model of AD by mediating the expression of miR-132 [278]. The exercise-associated improvement in the pro-neurogenic environment was associated with enhanced cognitive function in AD mice [278]. Additionally, volunteer exercise increases the proliferation of NPCs in mouse DG by down-regulating miR-135a [147]. Genetic suppression of miR-135a promotes NPC proliferation, leading to enhanced hippocampal neurogenesis [147]. The beneficial impact is linked to the modulation of 17 proteins, which are predicted targets of miR-135a in NPCs. Subsequent studies have identified a range of miRNAs, such as miR-34, miR-144-5p, miR-708-5p, miR-129–1-3p and miR-482, that are implicated in the exercise-induced enhancement of brain plasticity and cognitive function by stimulating hippocampal neurogenesis [279, 284, 285]. Interestingly, Improta-Caria et al. conducted a novel pooled analysis to evaluate the relationship between exercise-associated changes in miRNAs and AD symptoms by comparing the lists of miRNAs that are altered in AD and by exercise [286]. The analysis yielded 27 overlapping miRNAs between AD and exercise, of which 10 miRNAs displayed opposite expression patterns in AD versus exercise, which are mainly involved in cell metabolic, proteolytic, and inflammatory pathways [286]. miRNAs are a particular cluster of compounds that play a crucial role in the interaction between exercise and neurological disorders. miRNAs regulate exercise-associated neurobiological effects by binding a variety of target genes that are involved in the regulation of neuronal plasticity and function [280, 281, 287]. Recent studies have shown great interest in the therapeutic potential of miRNAs as exercise mimetics for brain disorders. Accordingly, a number of miRNAs have been identified as exercise mediators in various rodent models of neurological diseases, which may have the potential for treating brain disorders [283, 286, 288–291].

Considering the essential role of miRNAs in exercise and brain health, several studies have developed novel therapeutic approaches targeting miRNAs and relevant signalling to treat or delay neuropathological processes. MiR-146 is among the most investigated, commonly dysregulated miRNAs with known or potential roles in the neuroimmune interface in AD [292]. MiR-146 is highly expressed in microglia [293–295] and miR-146 knock-out mice do not show effective microglial phagocytosis in response to lipopolysaccharide (LPS), indicating that miR-146 is essential for microglial response to neuropathological stimuli [296]. Exercise can alter the expression and function of miR-146 in various tissues [297–299]. To test the effects of miR-146a on AD pathology, Mai et al. intranasally administered a miR-146a agomir (M146AG) to transgenic AD mice. They found alleviation of amyloid and tau pathologies and improvement of neuroinflammation and cognitive function [300]. MiR-146 plays an important role in promoting hippocampal neurogenesis, and restoring the miR-146 level improves AHN and cognition [301]. Moreover, neuroinflammation is considered a major contributor to the impairment of AHN in various neurodegenerative diseases [47, 302, 303]. Therefore, reducing neuroinflammation linked to miR-146 expression may alleviate the impairment of AHN in these neurological disorders. MiR-23b-3p is recognised as a robust modulator of α-synuclein, showing therapeutic potential in treating PD pathology [276]. MiR-23b-3p is significantly downregulated in PD patients as well as in NSCs and rats treated with 6-hydroxydopamine. Cai et al. [276] found that the miR-23b-3p mimic decreased α-synuclein mRNA by 65%, while the miR-23b-3p inhibitor increased α-synuclein by 1.4 fold. Other miRNAs such as miR-155, miR-21, miR-7, and miR-129 also show therapeutic potential for brain disorders such as AD, PD, and stroke in animal studies [304–308]. Despite the therapeutic potentials by targeting miRNAs, there are challenges in the use of those compounds to treat neurological disorders. Current evidence may shed some light on the directions for future research to elucidate the roles of these molecules in mediating the effects of exercise on the brain.

### Glycosylphosphatidylinositol (GPI)–specific phospholipase D1 (Gpld1)

Apart from the above-mentioned exercise mimetics, several novel molecules have been tested recently and shown potential in mimicking the effects of exercise on brain plasticity and function [34, 37] (Fig. 3). Free running leads to elevated adult neurogenesis, up-regulation of BDNF expression, and enhanced hippocampal-dependent memory performance in aged rodents. These effects could be transferred to sedentary aged mice via intravenous injection (8 times for 3 weeks) of circulating blood factors in plasma isolated from the aged running rodents [37]. Liquid chromatography–tandem mass spectrometry and functional enrichment analysis identified a liver-originated, exercise-stimulated circulating factor, Gpld1, to correlate with the exercise-induced memory improvement [37]. In addition, increased plasma concentrations of Gpld1 were also identified among active older adults compared to sedentary age-matched individuals. Hepatic overexpression of Gpld1 markedly increased circulating GPI levels and was sufficient to enhance hippocampal neurogenesis and cognitive performance in aged rodent hippocampus by triggering signalling pathways downstream of GPI-targeted substrate cleavage [37]. This study identifies a liver–brain communication by which the hepatic-derived blood compound confers effects of exercise to aged mice, raising the potential of manipulating GPI expression to recapitulate the beneficial effects of exercise.

#### Clusterin

Mice that received ‘runner plasma’ collected from exercised mice show a markedly increased number of neural stem/progenitor cells and DCX^+^ neuroblasts, enhanced contextual learning and memory function, and decreased expression of inflammatory genes in the hippocampus [34]. Interestingly, the capacity of blood factors from ‘runner plasma’ to recapitulate the exercise effects shows an exercise duration-dependent manner. The plasma collected from mice with a running-wheel exercise for 28 days is more likely to replicate ‘exercise effects’ compared to plasma from mice with wheel-running for 7 or 14 days [34]. The ‘runner plasma’ shows a potent anti-inflammatory effect on neural response to LPS in the hippocampus, and subsequently, an anti-inflammatory factor–clusterin was identified. As a putative ‘blood component’, depletion of clusterin largely abolished the anti-neuroinflammatory effect of runner plasma, whereas treatment with recombinant clusterin inversed the cerebral expressions of immune and inflammatory transcripts in the mouse hippocampal endothelial cells with inoculation of LPS [34]. Moreover, serum levels of clusterin were markedly elevated after a 6-month exercise intervention in the patients with amnestic mild cognitive impairment compared to basic values before interventions, demonstrating the possibility of translating exercise effects to humans via management of clusterin [34]. While this study proposed clusterin as a key factor contributing to the positive effect of exercise on AHN and cognition, the direct impact of clusterin on hippocampal neurogenesis was not determined. This underscores the need for future investigations to address specifically this issue.

Recently, a meta-analysis encompassing a total of 28 studies found that the levels of clusterin are significantly elevated in both the plasma and the brain tissues of individuals with dementia compared to healthy control subjects [309]. Similar increases in clusterin concentrations were also observed in individuals with PD and dementia with Lewy bodies [310]. These data suggest that changes in clusterin levels may play an essential role in mediating the pathology of those diseases. Early research has indicated that overexpression of α-synuclein in SH-SY5Y human neuroblastoma cells increases clusterin expression, whereas decreasing clusterin expression aggravates α-synuclein deposition, highlighting the role of clusterin in modulating α-synuclein accumulation [311]. Further research has found that clusterin interacts with the α-synuclein complex in the lysosomal systems for α-synuclein degradation [312]. Additionally, clusterin can directly interact with Aβ and tau proteins, preventing the formation of Aβ oligomers and fibril tau and promoting their clearance [313, 314]. Clusterin shows multiple impacts on neuronal plasticity, such as promoting the survival and differentiation of neurons derived from NPCs [315] and increasing glutamatergic synaptic transmission and dendritic spine density [312]. The protective role of clusterin in facilitating the removal of misfolded proteins like α-synuclein, Aβ and tau proteins may offer potential therapeutic strategies, such as physical exercise or agents targeting clusterin expression, for alleviating neuropathology and enhancing brain plasticity and function. However, the function of clusterin in regulating Aβ and tau in AD pathology is complicated and may differ across the different stages of the disease [312, 316]. This indicates that the present understanding of the precise mechanisms mediating the interaction between clusterin and the pathology of misfolded proteins is not fully addressed, highlighting a critical area for future research endeavours. Nonetheless, as suggested by Palihati and colleagues [312], clusterin plays a crucial role in regulating neuronal plasticity and cognitive functions associated with neurodegenerative disorders; thus, therapeutic strategies targeting this protein could be a promising approach to managing these disorders.

#### Serotonin

Serotonin has attracted particular interest as studies have revealed increased adult neurogenesis in the hippocampus after treatment of selective serotonin reuptake inhibitors, which is regarded as indispensable for antidepressant effects [317, 318]. Research has shown a decrease in serotonin expression in the brains of AD patients compared to matched controls, with similar reductions observed in peripheral and CSF levels [319, 320]. Furthermore, dysregulated plasma serotonin levels have been linked to non-motor symptoms in PD patients [321, 322], suggesting a regulatory role for serotonin dysregulation in neurodegenerative diseases. Moreover, serotonin-mediated adult neurogenesis is involved in exercise-related beneficial effects on brain plasticity and function [323, 324]. Tryptophan hydroxylase 2-KO mice, a rodent model absolutely deficient in brain serotonin, display impaired NSC proliferation in response to exercise [324]. Additionally, *Htr3a*^*−/−*^ mice with deletion of 5-HT type 3A receptor subunit, show resistance to exercise-induced hippocampal neurogenesis, whereas administration of serotonin receptor agonist increases newly generated neurons in the rodent hippocampus, which partly recapitulates exercise effects [325]. Those data reinforce the crucial role of serotonin-induced hippocampal neurogenesis in mediating the beneficial effects of antidepressant agents and exercise interventions [317, 318, 324].

#### 5-Aminoimidazole-4-carboxamide ribonucleotide (AICAR): mimetics targeting metabolic pathway

The AMP-activated protein kinase (AMPK)–Sirtuin 1–PGC-1α–PPARγ signalling network is an important metabolic pathway that regulates energy consumption and mitochondrial function in response to exercise (Fig. 3). Pharmacological targeting of metabolic networks has generated several molecular agents, such as AICAR, metformin and GW501516. AICAR is an analogue of AMP that can affect multiple organ functions. It mediates a plethora of metabolic processes partly via recapitulating exercise effects, including up-regulating glucose transport protein (GLUT)-4 expression, hexokinase activities, muscle mitochondrial homeostasis and VEGF expression, as well as ameliorating inflammatory process [326–328]. Its featured function is the capacity to replicate, in part, exercise effects on brain health. Administration of AICAR (500 mg/kg) in adult rodents for 1 week led to increased hippocampal neurogenesis and PS performance [329]. Even in old mice (2 years), the treatment with AICAR resulted in improvement of memory and motor coordination [329]. Moreover, the administration of AICAR could promote the expression of insulin-degrading enzymes and reduce Aβ deposition in mice with AD, resulting in enhanced spatial learning and recognition performance [330]. Additionally, AICAR treatment also alleviates AD-like pathological changes including biochemistry and cognitive function in rodent models [331]. The beneficial impact of AICAR in the context of AD may be correlated to its anti-neuroinflammatory effects. Ayasolla et al. revealed that AICAR administration has inhibitory effects on LPS/Aβ-induced inflammatory response by reducing the production of pro-inflammatory cytokines (such as TNF-α, IL-1β, and IL-6) and attenuating ROS generation and glutathione depletion in glial cells [332].

#### Metformin

Metformin, a key compound from the biguanidine class, is among the primary drugs prescribed to patients with type 2 diabetes to control hyperglycemia by up-regulating GLUT-4 (glucose transporter 4) expression and membrane translocation in skeletal muscle and adipose tissues [333, 334]. Administration of metformin (200 mg/kg per day, 38 days) markedly promoted adult neurogenesis and spatial memory function in rodent hippocampus [335]. Moreover, in a rodent stroke model, 30-day treatment of metformin (50 mg/kg per day) led to memory improvement and increased adult-born neurons in the hippocampus via elevating AMPK activity [336]. It suggests that the exercise-induced favourable impact on brain plasticity and function could be recapitulated in part by metformin administration. The relationship between long-term metformin use and the risk of AD has been extensively investigated. Several studies have shown that metformin use may reduce the risk of AD and improve cognitive function in diabetes patients [337, 338]. For example, Hsu et al. [339] matched 800 diabetic patients who developed dementia with 3200 controls. They reported that metformin use reduced the risk of dementia (adjusted odds ratio [AOR] 0.46; 95% CI 0.35–0.61), especially AD (AOR 0.38; 95% CI 0.25–0.58), compared with controls without metformin use. However, the results are not always consistent. Using a UK primary care database, Imfeld et al. [340] reported that metformin use increased the risk of dementia (AOR 1.23, 95% CI 1.13–1.34), especially AD (AOR 1.29, 95% CI 1.15–1.44), compared with non-use of metformin. The underlying reasons for the discrepancy across studies may include variations in study design, population characteristics, confounding factors, and outcome measures. For instance, Ng et al. [341] performed a meta-analysis of observational studies and reported that metformin use reduced the risk of AD in patients with diabetes (risk ratio [RR] 0.76, 95% CI 0.63–0.92), but not in patients without diabetes (1.05, 95% CI 0.76–1.46). Since metformin use has been shown to reduce Aβ deposition and abnormal tau phosphorylation, alleviate inflammation, increase insulin sensitivity, and enhance neurogenesis [342–345], all of which are key mechanisms of AD, the relationship between metformin use and AD risk warrants further investigation.

#### GW501516 and cautions on metabolic pathway mimetics

Other compounds targeting AMPK pathways, such as GW501516, also show neuroprotective effects against neuropathology in a murine model of PD [346]. The neuronal protective effects of this compound may involve its anti-inflammatory effects since GW501516 treatment could reduce the interferon-α-induced inflammation in brain cells [347]. Moreover, GW501516 administration (5 mg/kg per day, for 7 days) could markedly promote memory function and hippocampal neurogenesis in young female mice [348]. Collectively, those compounds could replicate, to some degree, exercise effects and likely have clinical potential in counteracting neurodegeneration and cognitive dysfunction. However, there are some essential questions or limitations that need to be addressed for those compounds in treatment. For example, due to the low permeability across BBB, the precise doses for AICAR and GW501516 treatment are still not established [349]. Furthermore, long-term administration of those compounds may induce side effects. For example, mice with long-term AICAR treatment do not show expected enhancement in markers or genes associated with neurogenesis and cognition [329], and there might be unintended or off-target effects due to the varying distribution of AMPK receptor subtypes in different tissues of the body [350]. Moreover, long-term treatment with metformin may induce an adverse impact on cognition [340, 351]. Additionally, some side effects have been reported for the treatment of GW501516, such as gastrointestinal discomfort, muscle cramps, joint pains, and cancer risk [352, 353]. Therefore, while these compounds show promise, more research is needed to address the questions or limitations regarding the dosage, long-term safety, and potential side effects before they can be considered viable treatments for enhancing AHN and cognition.

## Conclusions

In the ageing society, there is a growing burden of diseases and medication or healthcare costs. Novel, effective approaches for dealing with those issues are urgently needed. An active lifestyle is a preferred choice for improving body and brain health. Compounds derived from runner plasma, have proven valuable in achieving this goal, especially for people with limited mobility due to diseases, injuries, or ageing-associated frailty.

Blood components frequently show particular effects, which are somewhat different from the general impact of exercise. The influence of certain specific molecules (such as BDNF and irisin) on hippocampal neurogenesis and cognition presents great interest due to the fact that some neurodegenerative disorders (such as AD and PD) are regarded as incurable. Additionally, the tested molecules are often administered systematically and show marked cerebral effects [34, 36, 37], such as neurogenesis, synaptic plasticity, and cognitive function, without crossing the BBB. Moreover, exercise mimetics have one unique advantage: some biomarkers are available in a range of therapeutic products, such as probiotics and prebiotics.

However, there are some questions that need to be addressed in future studies. Current research methods are primarily conducted on animal models; therefore, the data obtained from these studies should be applied with caution to patients with neurodegenerative disorders. For instance, AHN has been demonstrated in animal hippocampi, but efficient methods for determining AHN in humans are currently lacking. Consequently, assessing the impact of exercise on AHN in patients with neurodegenerative diseases remains challenging. Moreover, the optimal exercise protocol for enhancing AHN and cognitive function has not yet been established, including the types and duration of exercise interventions. Considering the diverse pathological origins of neurodegenerative diseases, the specific exercises designed to address these varying pathologies and enhance AHN could potentially vary across different conditions. Therefore, the urgent priority is to construct disease-specific, comprehensive models (at different levels: molecule, cell, and system) that can help elucidate the underlying mechanisms of how exercise impacts the brain. Moreover, the following step is to discriminate the causal from correlative compounds involved in the molecular process of brain response to exercise intervention, and those mediating molecules could be targets for pharmacological and clinical development. The treatment of neurological disorders with exercise mimetics is still in its infancy, and the optimal dose, timing, and duration of compounds are not well established. The safety and efficacy of exercise mimetics in humans need to be evaluated in clinical trials.

## Data Availability

Not applicable.

## Abbreviations

AHN: Adult hippocampal neurogenesis
HD: Huntington's disease
NSC: Neural stem Cell
DG: Dentate gyrus
DGCs: Dentate gyrus granule cells
snRNA-seq: Single-nucleus RNA sequencing
BrdU: Bromodeoxyuridine
PSANCAM: Polysialic acid-neural cell adhesion molecule
APP: Amyloid precursor protein
PS1: Presenilin 1
CSF: Cerebrospinal fluid
IGF-1: Insulin-like growth factor-1
GABA: Gamma-aminobutyric acid
5HT: Serotonin
DA: Dopamine
ACh: Acetylcholine
FGF-2: Fibroblast growth factor-2
TrkB: Tropomyosin receptor kinase B
FNDC5: Fibronectin type III domain containing 5
IL-6: Interleukin 6
BDNF: Brain-derived neurotrophic factor
AD: Alzheimer's disease
PPARγ: Peroxisome proliferator-activated receptor gamma
PGC1α: Peroxisome proliferator-activated receptor gamma coactivator 1 alpha
PD: Parkinson's disease
TNF-α: Tumor necrosis factor alpha
CNS: Central nervous system
SCFAs: Short-chain fatty acids
BBB: Blood-brain barrier
miRNAs: MicroRNAs
Gpld1: Glycosylphosphatidylinositol specific phospholipase D1
LPS: Lipopolysaccharide
AMPK: AMP-activated protein kinase
AICAR: 5-Aminoimidazole-4-carboxamide ribonucleotide
VEGF: Vascular endothelial growth factor
